# The Chemokine Receptor CXCR6 Evokes Reverse Signaling via the Transmembrane Chemokine CXCL16

**DOI:** 10.3390/ijms18071468

**Published:** 2017-07-08

**Authors:** Vivian Adamski, Rolf Mentlein, Ralph Lucius, Michael Synowitz, Janka Held-Feindt, Kirsten Hattermann

**Affiliations:** 1 Department of Neurosurgery, University Medical Center Schleswig-Holstein UKSH, Campus Kiel, D-24105 Kiel, Germany; Vivian.adamski@uksh.de (V.A.); Michael.synowitz@uksh.de (M.S.); Janka.held-feindt@uksh.de (J.H.-F.); 2 Department of Anatomy, University of Kiel, D-24118 Kiel, Germany; rment@anat.uni-kiel.de (R.M.); rlucius@anat.uni-kiel.de (R.L.)

**Keywords:** chemokine, chemokine receptor, reverse signaling, cellular communication, brain tumor, glioma, tumor cell migration

## Abstract

Reverse signaling is a signaling mechanism where transmembrane or membrane-bound ligands transduce signals and exert biological effects upon binding of their specific receptors, enabling a bidirectional signaling between ligand and receptor-expressing cells. In this study, we address the question of whether the transmembrane chemokine (C-X-C motif) ligand 16, CXCL16 is able to transduce reverse signaling and investigate the biological consequences. For this, we used human glioblastoma cell lines and a melanoma cell line as in vitro models to show that stimulation with recombinant C-X-C chemokine receptor 6 (CXCR6) or CXCR6-containing membrane preparations induces intracellular (reverse) signaling. Specificity was verified by RNAi experiments and by transfection with expression vectors for the intact CXCL16 and an intracellularly-truncated form of CXCL16. We showed that reverse signaling via CXCL16 promotes migration in CXCL16-expressing melanoma and glioblastoma cells, but does not affect proliferation or protection from chemically-induced apoptosis. Additionally, fast migrating cells isolated from freshly surgically-resected gliomas show a differential expression pattern for CXCL16 in comparison to slowly-migrating cells, enabling a possible functional role of the reverse signaling of the CXCL16/CXCR6 pair in human brain tumor progression in vivo.

## 1. Introduction

Cellular communication is frequently mediated by more or less specific binding of a ligand to its corresponding receptor, exerting intracellular signaling cascades and downstream effects in the receptor-expressing cell. However, transmembrane or membrane-bound ligands can also serve as signaling “receptors” and thus enable a bidirectional cellular communication. This signaling mode is termed “reverse signaling” and has so far been described for members of some (super)families of transmembrane ligands including the tumor necrosis factor-α (TNFα) superfamily, the ephrin ligand family and the semaphorins; for a review, see [1,2,3]. Reverse signaling depends on the intracellular domains of the ligands and/or associated molecules. This intracellular communication is involved in immune regulation and modulation [1,4,5], development and maintenance of the nervous system including axon guidance and synaptic plasticity [2,3,6,7], bone remodeling [8] and vascular morphogenesis and angiogenesis [9].

Recently, we were able to report another alternative signaling mode that is mediated via the transmembrane chemokines CXCL16 and CX3CL1 (chemokine (C-X3-C motif) ligand 1). In this process, upon shedding by matrix metalloproteinases (a disintegrin and metalloproteinase (ADAM) 10 and ADAM17), the chemokine domain can be released from the transmembrane stack [10,11,12], binds to the transmembrane form and elicits intracellular extracellular signal-regulated kinase ½ (ERK1/2, p42/p44) and Akt signaling followed by downstream proliferative and anti-apoptotic effects in glioma cell lines and primary human meningioma cells [13,14]. Apart from this novel signaling mode, the soluble forms of CXCL16 and CX3CL1, of course, evoke effects via their known receptors. CXCL16 is a ligand for the chemokine receptor and HIV (human immunodeficiency virus) co-receptor CXCR6/Bonzo [15] and recruits immune cells, e.g., in rheumatoid arthritis [16]. However, CXCL16 and/or CXCR6 are also overexpressed in several types of tumors, including breast, prostate and gastrointestinal cancers, and benign and malignant tumors of the nervous system [17,18,19,20,21,22]. Within these tumors, the CXCL16/CXCR6 axis plays a multifaceted role by promoting proliferation and migration of tumor cells [17,18,19,21] and attraction and modulation of immune cells supporting immune-mediated tumor control [23,24,25].

Thus, regarding the facts that (1) reverse signaling via transmembrane ligands has been reported for a considerable number of ligand-receptor pairs and (2) we recently could show that transmembrane CXCL16 can transduce signals via its intracellular domain upon binding of its soluble form (“inverse signaling”), we wondered if reverse signaling may also take place in the interaction between transmembrane CXCL16 and its known receptor CXCR6.

To investigate intracellular signaling of CXCL16 upon stimulation with CXCR6, initially, we used human glioblastoma cell lines (known to express transmembrane CXCL16, but not CXCR6) and applied CXCR6 in different forms. The specificity of reverse signaling was proven by silencing experiments, as well as by transfection experiments using a CXCL16-negative, CXCR6-negative melanoma cell line to investigate intracellular signaling and biological effects upon stimulation with CXCR6.

## 2. Results

### 2.1. Expression of CXCL16 and CXCR6 in Native and Stably-Transfected Human Tumor Cell Lines

**Figure 1:**
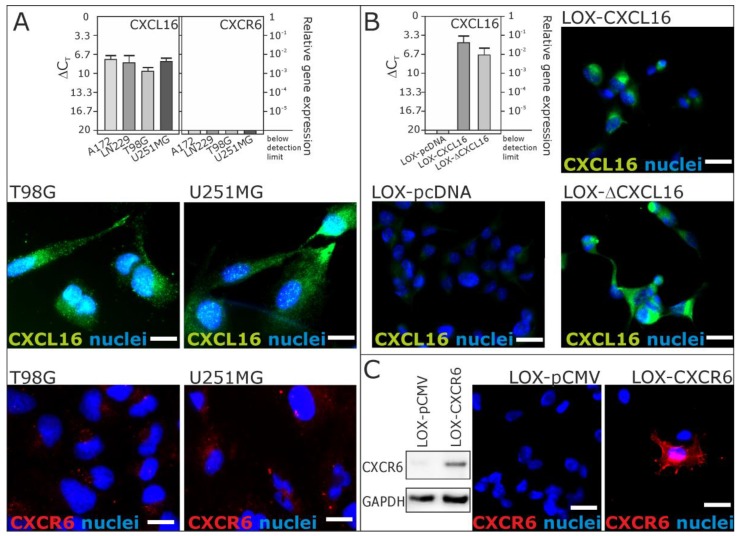
Expression of CXCL16 and CXCR6 mRNA and protein in glioblastoma cells and stably transfected LOX melanoma cell clones by quantitative reverse transcription polymerase chain reaction (qRT-PCR) and immunocytochemistry (ICC). (**A**) Expression of CXCL16 and CXCR6 was investigated in glioblastoma cell lines A172, LN229, T98G and U251MG (for biological independent results of A172 and T98G, compare also [13]). CXCL16 was detected at moderate to high extends, whereas CXCR6 was undetectable or yielded just background staining; (**B**) expression of CXCL16 was investigated in clones from natively CXCL16-negative, CXCR6-negative LOX melanoma cells. While the LOX-pcDNA clone was CXCL16 negative, the LOX-CXCL16 clone showed CXCL16 at the mRNA and protein level. A C-terminally truncated version of CXCL16 (in LOX-ΔCXCL16 cells) was also detectable at the mRNA and protein level (for verification of truncation, see [13]); (**C**) Expression of CXCR6 was investigated in LOX melanoma cell clones. While the LOX-pCMV clone was CXCR6 negative, a LOX-CXCR6 transfected clone yielded positive staining for CXCR6 and a specific signal at about 43 kDa in Western blot experiments. Values of qRT-PCR are shown as Δ*C*_T_, meaning that a 3.33 higher Δ*C*_T_ indicates a 10-fold lower mRNA expression. *n* = 3 independent experiments; examples shown for immunocytochemistry. Scale bars indicate 20 µm, respectively.

From our recent investigations, we know that CXCL16 is highly expressed in different human gliomas, while the corresponding receptor CXCR6 is restricted to a small subset of glioma cells with stem cell characteristics [20]. To investigate a putative reverse signaling mediated by transmembrane CXCL16, we used CXCL16-positive and CXCR6-negative glioblastoma cell lines. We verified the expression of CXCL16 and the lack of CXCR6 in human glioblastoma cell lines A172, LN229, T98G and U251MG on mRNA level by quantitative reverse transcription polymerase chain reaction (qRT-PCR) and on protein level by immunocytochemistry (ICC) for cell lines used in the following sections (Figure 1A; compare also [13] for independent results on T98G and A172).

To prove specificity, we used stable transfected LOX melanoma cell clones. LOX melanoma cells do not endogenously express CXCL16, nor CXCR6, and so, we generated LOX clones expressing transmembrane CXCL16 (LOX-CXCL16) or a C–terminally truncated version of transmembrane CXCL16 (LOX-ΔCXCL16) and a clone from the empty expression vector (LOX-pcDNA) [13]. To verify the expression of CXCL16 (and CXCR6) of the LOX cell clones used for the following assays, we performed qRT-PCR and immunocytochemistry (Figure 1B). Additionally, we used LOX melanoma cells to generate stable clones expressing CXCR6 (LOX-CXCR6). For controls, the empty control vector was inserted (LOX-pCMV), and we confirmed CXCR6 expression by immunocytochemistry and Western blot (Figure 1C).

### 2.2. Recombinant CXCR6 Induces ERK1/2 Phosphorylation via CXCL16 in Glioblastoma Cells

**Figure 2:**
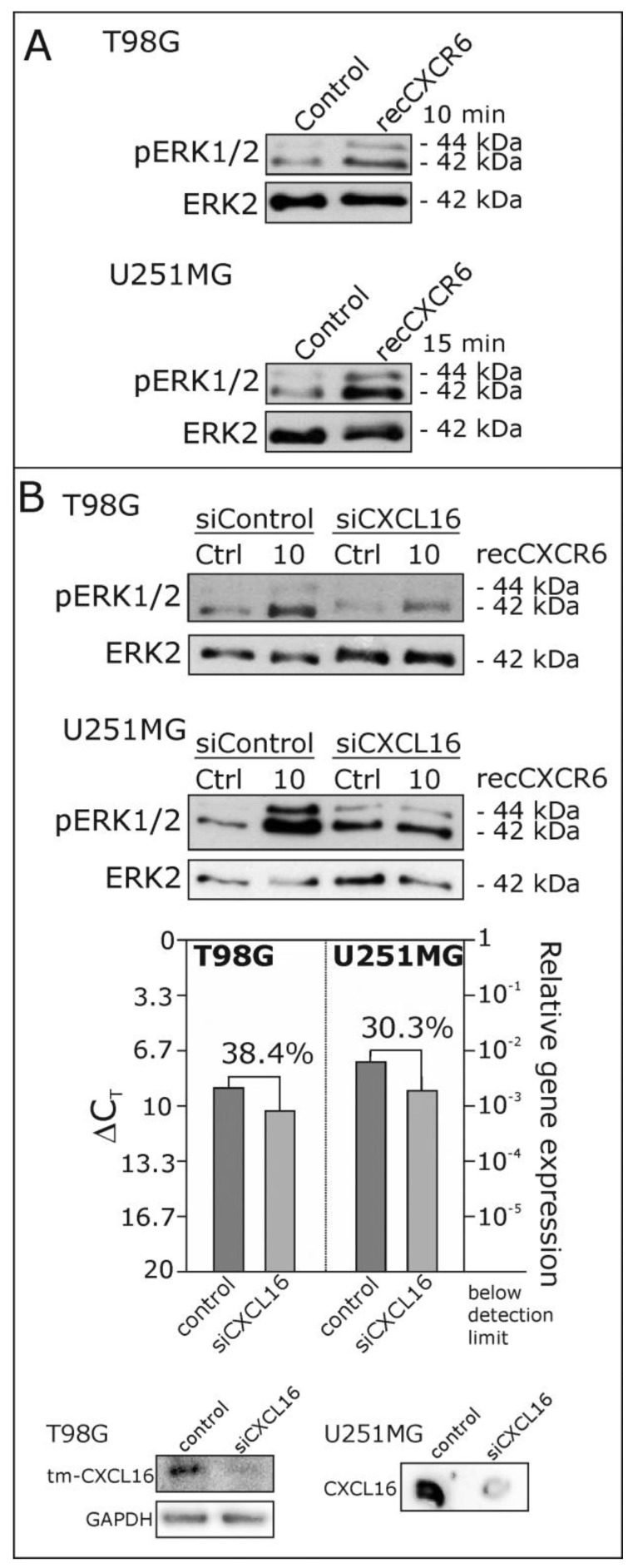
Phosphorylation of the extra cellular-regulated kinases ERK1/2 upon stimulation with recombinant CXCR6 in glioblastoma cells. (**A**) T98G and U251MG glioblastoma cells were stimulated with 25 ng/mL recombinant (rec) CXCR6 for 10 or 15 min, respectively, and phosphorylation of ERK1/2 was investigated by Western blot; equal loading was ensured by reprobing of the membranes with antibodies for the non-phosphorylated kinase ERK2. Stimulation with recombinant CXCR6 yielded a clear phosphorylation signal for both cell lines; (**B**) when CXCL16 expression was reduced in T98G and U251MG cells to 30–40% by CXCL16-specific siRNA (siCXCL16) as proven by qRT-PCR and Western or dot blotting, ERK1/2 phosphorylation after 10 minutes of stimulation with recombinant CXCR6 was clearly diminished in comparison to control siRNA transfections. Examples of *n* = 3 independent experiments.

To investigate a putative reverse signaling of transmembrane CXCL16 upon binding of its known receptor CXCR6, for the first approach, we used different CXCL16-positive, CXCR6-negative glioblastoma cell lines and stimulated them with 25 ng/mL recombinant CXCR6 for 10 or 15 min. As shown in Figure 2A, this stimulation yields a phosphorylation of ERK1/2 in glioblastoma cells (T98G, U251MG). This effect drastically decreased when glioblastoma cells were transfected with siRNAs specifically targeting CXCL16 prior to stimulation with recombinant CXCR6 (Figure 2B, in comparison to control siRNA transfections), indicating a specific signaling mechanism via CXCL16. The efficiency of siRNA-mediated reduction of CXCL16 was proven by qRT-PCR and immunoblotting for each independent experiment (Figure 2B, lower part).

### 2.3. Signaling of Recombinant and Membrane-Expressed CXCR6 Depends on the Expression of Intact Transmembrane CXCL16

**Figure 3:**
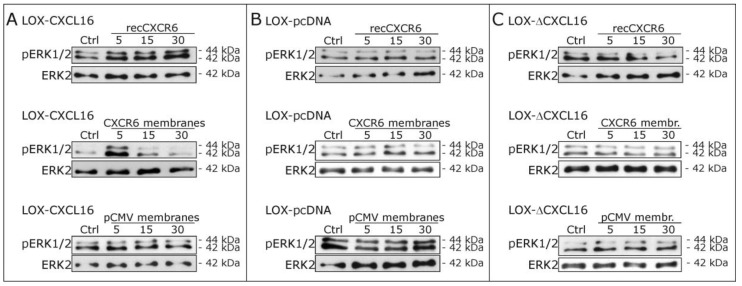
Phosphorylation of ERK1/2 upon stimulation with recombinant CXCR6 or membrane preparations of CXCR6-expressing and control LOX clones. (**A**) In LOX-CXCL16 clones, stimulation with 25 ng/mL recombinant (rec) CXCR6 (upper panel), as well as with membranes from CXCR6-expressing LOX cells (CXCR6 membranes, 5 µg/mL membrane preparation, middle panel) induced a robust phosphorylation of ERK1/2, while stimulation with control membranes lacking CXCR6 (pCMV membranes, lower panel) failed to activate ERK1/2; (**B**) in LOX-pcDNA cells that are CXCL16-negative and CXCR6-negative, stimulation with neither recombinant CXCR6, nor CXCR6 membranes, nor pCMV membranes yielded ERK1/2 phosphorylation; (**C**) LOX-ΔCXCL16 cells lacking the intracellular domain of the transmembrane CXCL16 also did not show any activation of the ERK1/2 signaling pathway upon stimulation with recombinant or membrane expressed CXCR6. Examples of *n* = 3 independent experiments.

In the next step, we stimulated stably CXCL16 expressing LOX melanoma cells (LOX-CXCL16) with recombinant CXCR6 and observed again an activation of ERK1/2 (Figure 3A, upper part). Additionally, we extracted membranes from stably CXCR6 expressing LOX cells (LOX-CXCR6) and used these for stimulations yielding also the activation of ERK1/2 signaling, while stimulation with control membranes from LOX-pCMV failed to induce phosphorylation. As a control, we repeated stimulation experiments with LOX-pcDNA control cells that are negative for CXCL16 (and also CXCR6), and neither recombinant, nor membrane expressed CXCR6 could activate ERK1/2 signaling (Figure 3B). Furthermore, we stimulated LOX melanoma cells stably expressing a CXCL16 variant that lacks the cytoplasmic tail due to truncation (LOX-ΔCXCL16) with recombinant and membrane expressed CXCR6, as well as control membrane fractions and did not observe any ERK1/2 phosphorylation either (Figure 3C). As a further control, to exclude unspecific reaction of a recombinant receptor preparation, we stimulated LOX-CXCL16 and LOX-pcDNA cells also with recombinant CX3CR1. CX3CR1 is the receptor for the transmembrane chemokine CX3CL1, which is not expressed by LOX clones. CX3CR1 does not bind to CXCL16, so that recCX3CR1 may serve as an unrelated recombinant receptor control. Accordingly, stimulation with recCX3CR1 did not yield any activation of the ERK1/2 pathway (compare Figure S1).

These results indicate that signaling upon CXCR6 stimulation specifically depends on the expression of CXCL16 including its intracellular domain and may physiologically occur upon exposition of transmembrane CXCL16 to CXCR6-expressing membranes.

### 2.4. Biological Effects of Reverse Signaling via CXCL16

**Figure 4:**
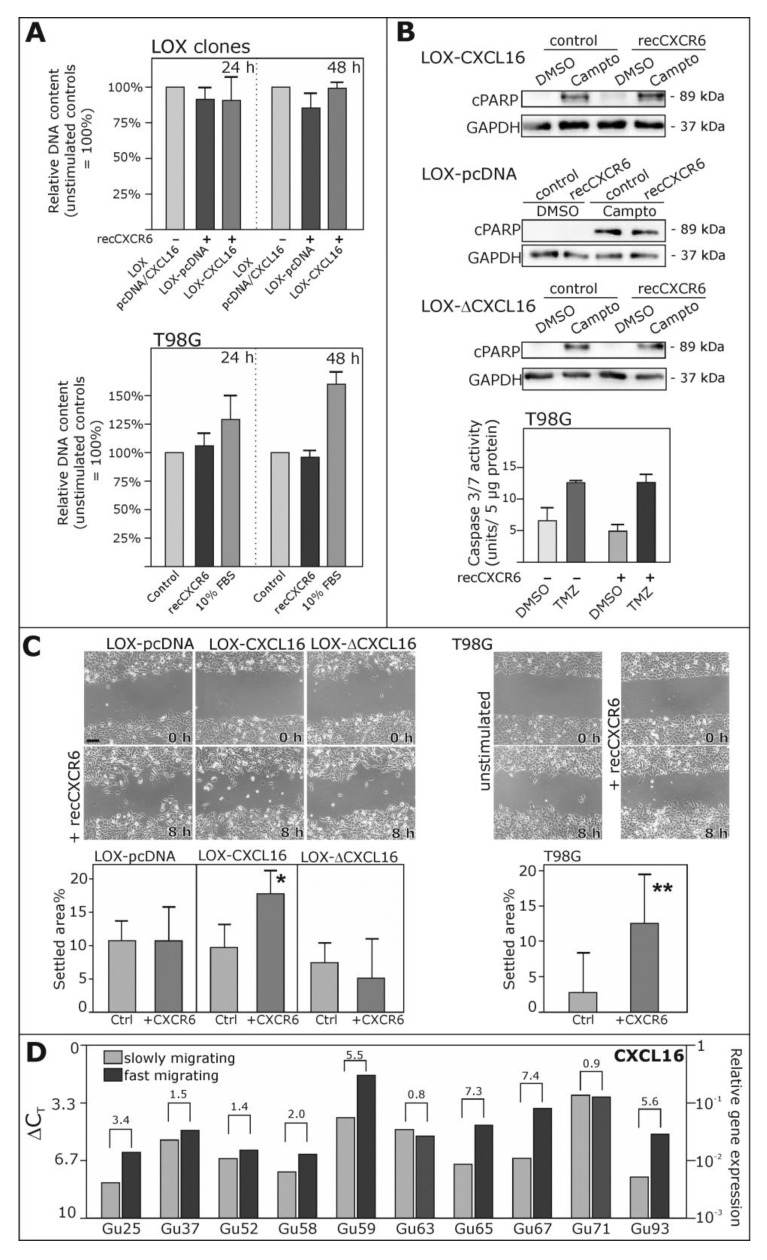
Biological effects of reverse signaling via the CXCR6-CXCL16-axis. (**A**) To investigate effects on proliferation, DNA contents were measured in LOX-CXCL16 and as a control in LOX-pcDNA cells stimulated (or not) with 50 ng/mL recombinant (rec) CXCR6 for 24 or 48 h (upper part). Corresponding experiments were also performed with T98G glioblastoma cells (lower part); 10% fetal bovine serum (FBS) served as the positive control for proliferation. Unstimulated controls were set to 100%, respectively, and stimulation with CXCR6 did not yield any significant induction or reduction of DNA content. Mean ± SD from *n* = 3 independent experiments; (**B**) apoptosis was induced with 0.1 µg/mL camptothecin (Campto), in comparison to equal volumes of solvent control dimethylsulfoxide (DMSO) for 18 h in LOX-CXCL16, LOX-pcDNA and LOX-ΔCXCL16 cells or for 48 h in T98G glioblastoma cells, and simultaneous stimulation with 50 ng/mL recCXCR6 did not reduce cleavage of PARP (cPARP) as detected by Western blot or caspase 3/7 activity as determined by fluorimetric measurement of substrate cleavage, both indicating apoptosis. For Western blotting, equal loading was ensured by reprobing of the membrane with a glyceraldehyde 3-phosphate dehydrogenase (GAPDH)-specific antibody. Examples (Western blot) or mean values (caspase activity) of *n* = 3 independent experiments; (**C**) to investigate migration, scratch assays were performed with LOX clones LOX-CXCL16, LOX-pcDNA and LOX-ΔCXCL16 or T98G glioblastoma cells stimulated with 50 ng/mL recCXCR6 or left unstimulated for controls. Scratch areas were measured at the beginning and after 8 h, and settled areas were determined as the percentage of the initial scratch area. Stimulation with 50 ng/mL CXCR6 promotes migration of LOX-CXCL16 and T98G cells, but not LOX-pcDNA or LOX-ΔCXCL16 cells. Mean ± SD from *n* = 4 independent experiments; exemplary images are shown with equal magnifications, respectively; scale bar indicates 50 µm; * *p* < 0.05, ** *p* < 0.01; (**D**) fast migrating glioblastoma cells from freshly-dissected glioblastomas mostly show higher CXCL16 mRNA expression levels than the slowly migrating cells of the same tumor preparation. Δ*C*_T_ levels are shown in a logarithmic scale (a 3.33 higher Δ*C*_T_ value indicates a 10-fold lower mRNA expression), and numbers above the brackets indicate the (linearized) x-fold expression difference between fast and slowly-migrating cells of ten different primary and secondary glioblastoma samples.

To investigate which biological consequences might result from the reverse signaling of the CXCL16-CXCR6 axis, we first referred to the effects observed with inverse signaling in glioma cells [13] and tested the proliferative and anti-apoptotic effects in LOX-CXCL16 and corresponding control clones. However, in these CXCL16-transfected melanoma cells, we did not observe any regulation of proliferation upon stimulation with recombinant CXCR6 (Figure 4A), nor could we detect less cleavage of poly(ADP ribose) polymerase (PARP, Figure 4B) after induction of apoptosis with 0.1 µg/mL camptothecin. Additionally, we did not observe any proliferative or anti-apoptotic effects in endogenously CXCL16-expressing glioblastoma cells (Figure 4A,B).

Next, we investigated the migratory potential of LOX-CXCL16 cells in comparison to LOX-pcDNA (control) and LOX-ΔCXCL16 (C-terminally truncated) and of T98G glioblastoma cells in a scratch assay with or without stimulation with recombinant CXCR6 (Figure 4C). Here, we could show that CXCR6 stimulation enhanced the migration into the cell free area in a time span of 8 h for LOX-CXCL16 and T98G glioblastoma cells, while there was no significant difference in migration between unstimulated and CXCR6-stimulated cultures in LOX-pcDNA and LOX-ΔCXCL16 cells.

Interestingly, when we investigated the expression of CXCL16 in fast migrating in comparison to slowly-migrating cells isolated from freshly-dissected glioblastomas, as described previously [26], we could show that in most investigated glioblastomas, CXCL16 expression was elevated in fast migrating cells in comparison to slowly-migrating ones (Figure 4D), which may indicate that high CXCL16 levels might favor migratory potential in glioblastomas.

Summarizing, our data show that transmembrane CXCL16 transduces signals upon stimulation with its known receptor CXCR6, activating intracellular ERK1/2 signaling. This reverse signaling depends on the intracellular domain of CXCL16 and promotes migration in CXCL16-expressing melanoma and glioblastoma cells in vitro. Additionally, we could show that fast migrating glioblastoma cells isolated from freshly-dissected glioblastomas express CXCL16 at higher levels in comparison to slowly-migrating cells, giving a first hint that reverse signaling might also contribute to glioblastoma migration processes in vivo.

## 3. Discussion

Physiologically, transmembrane CXCL16 is among others expressed by immune and endothelial cells and can be induced in inflammatory conditions [11,15,16,27]. The chemokine domain is shed from the transmembrane protein by the matrix metalloproteinases ADAM 10 and 17 [10,11,12] and promotes trafficking of immune cells [15]. Additionally, CXCL16 has been shown to increase proliferation, e.g., of glial precursor cells [28] and endothelial cells [29]. Interestingly, the transmembrane form of CXCL16 mediates firm adhesion contacts between ligand and CXCR6 receptor-expressing cells indicating that also transmembrane CXCL16 may bind to CXCR6 and does not afford an activation of CXCR6 [27].

However, recently, we showed that CXCL16 can also induce signals independently from CXCR6 by a mechanism we termed inverse signaling. In this signaling mode, the chemokine domain of CXCL16 binds to the transmembrane form of CXCL16, induces intracellular ERK1/2 and Akt signaling and promotes proliferation and rescue from chemically-induced apoptosis [13,14]. In the present study, we demonstrated that the transmembrane form of CXCL16 may also transduce signals upon stimulation with CXCR6 resulting in the activation of ERK1/2 followed by increased migration in the ligand-bearing cell. Additionally, as previously shown for inverse signaling, reverse signaling and downstream effects depend on the intact intracellular domain of CXCL16. In glioblastomas, CXCR6 is expressed by a small subset of tumor cells with stem cell properties [20], so that direct cell contacts might enable reverse signaling via CXCL16.

Regarding effects via transmembrane ligands in a more general view, the reverse signaling is often involved in modulating the balance in dynamic changing systems and plasticity, like for example the Eph (erythropoietin-producing human hepatocellular receptors)/ephrin interactions in the formation and maintenance of synapses [2], in angiogenesis [9] and in bone remodelling [8], TNF family members as co-stimulators and direct effectors in the adaptive and innate immune system [1,30] and the semaphorins in a variety of processes including axonal guidance, angiogenesis and immune response [31]. These interactions often evoke cell cytoskeleton rearrangement and migratory processes and involve a multitude of signaling pathways including, e.g., the ERK1/2, Akt and STAT3 (Signal transducer and activator of transcription 3) pathways [6,9,32,33]. Apart from its role in physiological development and homeostasis, reverse signaling has also been described in tumor progression showing diverse effects, for example breast cancer-associated angiogenesis [34] and increased glioma cell motility via ephrin-B2 [35]. Interestingly, the semaphorin Sema5A has been shown to inhibit glioma cell motility [36], while this and other semaphorins seem to promote cancer growth and metastasis [31].

Thus, reverse signaling contributes to tumor biology in a multifaceted way. We were able to show now that the transmembrane chemokine CXCL16 can also mediate reverse signaling and promotes migration in the tumor context. In this line, we observed that fast migrating glioblastoma cells show higher CXCL16 expression levels in comparison to slowly-migrating cells of the same tumors.

## 4. Materials and Methods

### 4.1. Cell Cultures and Freshly-Isolated Glioma Cells

The human glioblastoma multiforme (GBM) cell lines A172 (ECACC 880624218), U251MG (ECACC 89081403; formerly known as U373MG), T98G (ECACC 92090213) and LN229 (ATCC-CRL-2611) were obtained from the European Collection of Cell Cultures (ECACC, Salisbury, UK) or the American Type Culture Collection (ATCC, Manassas, VA, USA) and cultured as described before [26]. Fast and slowly-migrating native human GBM cells were isolated as mentioned previously [26] and in accordance with the Helsinki Declaration of 1975 with approval of the ethics committee of the University of Kiel, Germany, after written informed consent of donors (file reference: D 408/14). For an overview of clinical data available for these samples, please refer to [26]. Different GBM cells were checked for purity by immunostaining with markers specific for GBM cultures (glial fibrillary acidic protein (GFAP) and fibronectin [37,38]; compare Figure S2) and for the absence of *Mycoplasma* contamination. LOX melanoma cells were a gift from Udo Schumacher, Department of Anatomy, University of Hamburg, Germany [39]. Cell lines’ identity was proven routinely by short tandem repeat profiling at the Department of Forensic Medicine (Kiel, Germany) using the Powerplex HS Genotyping Kit (Promega, Madison, WC, USA) and the 3500 Genetic Analyser (Thermo Fisher Scientific, Waltham, MA, USA).

### 4.2. Stable Transfected Cell Lines

Stable transfected LOX-pcDNA, LOX-CXCL16 and intracellularly truncated LOX-ΔCXCL16 clones were generated as described previously [13].

Expression vectors for CXCR6 (CXCR6 ORF with C-terminal GFP-tag in a pCMV backbone, pCMV6-CXCR6-GFP, RG206517) and pCMV (pCMV-AC-GFP, PS100010) were obtained from OriGene (Herford, Germany), and transfection of LOX melanoma cells (250,000 cells) was performed with TurboFect (Fermentas, Sankt Leon-Rot, Germany) in serum-free Dulbecco’s Modified Eagle’s Medium (dulbecco’s modified eagle’s medium (DMEM); Invitrogen, Carlsbad, CA, USA) without antibiotics using 3 µg of the respective expression vectors and 3 µL TurboFect in a total volume of 1 mL. After 6 h, cells were rinsed, and normal growth medium (RPMI + 10% fetal bovine serum (FBS)) was added. Successful transfection was controlled by immunocytochemistry and quantitative real-time PCR (qRT-PCR). Stable clones were generated by selection with 0.8 mg/mL G418 (Calbiochem, Merck Company, Darmstadt, Germany), and colonies were picked after 10–20 days, amplified and checked for expression by qRT-PCR and immunocytochemistry.

### 4.3. Immunocytochemistry

Glioblastoma cell lines and different stably-transfected LOX melanoma cells grown on glass cover slips were prepared as described before [40]. Cells were incubated with primary and secondary antibodies; nuclei were stained; and slides were analyzed using a Zeiss fluorescence microscope and a Zeiss camera (Zeiss, Oberkochen, Germany). Primary antibodies were anti-CXCL16 (1:200, 500-P200, rabbit; Peprotech, Hamburg, Germany) and anti-CXCR6 (1:100, MAB699, mouse; R&D Systems, Systems, Minneapolis, MN, USA). Primary antibodies were omitted for negative controls. As secondary antibodies, donkey anti-mouse or anti-rabbit IgGs labeled with Alexa Fluor 488 or Alexa Fluor 555 (1:1000; Invitrogen, Carlsbad, CA, USA) were used.

### 4.4. Reverse Transcription and Quantitative Real-Time PCR 

RNA of the different cell types was isolated with the TRIzol^®^ Reagent (Invitrogen, Carlsbad, CA, USA) or with the ARCTURUS^®^ PicoPure^®^ RNA Isolation Kit (Applied Biosystems, Waltham, MA, USA) according to the manufacturer’s instructions. DNase digestion, cDNA synthesis and qRT-PCR were performed as described before [38] using TaqMan primer probes (Applied Biosystems): CXCL16 (Hs00222859_m1), CXCR6 (Hs00174843_m1), glyceraldehyde 3-phosphate dehydrogenase (GAPDH) (Hs99999905_m1). Cycles of threshold (*C*_T_) were determined, and ∆*C*_T_ values of each sample were calculated as *C*_Tgene of interest_ − *C*_TGAPDH_. A ∆*C*_T_ of 3.33 corresponds to a 10-fold lower expression compared to GAPDH. For statistical analysis, the relative gene expression compared to GAPDH (2^−∆*C*T^) was employed. The induction of gene expression upon stimulation is displayed as relative gene expression; n-fold expression changes were calculated as ∆∆*C*_T_ values = 2^∆*C*^_T_^control−∆*C*^_T_^stimulus^.

### 4.5. RNAi Silencing

After cultivation of human glioblastoma cell lines in DMEM plus 10% FBS in 6-well dishes (180,000 cells/well) for 24 h, cells were transfected with siCXCL16 RNA (CXCL16 siRNA ID: s33808; 20 nM/well; Life Technologies, Darmstadt, Germany) dissolved in a mixture of Opti-MEM medium and lipofectamine (Life Technologies) for 5 h as described before [13]. In parallel, a transfection with silencer select negative control siRNA (Life technologies) was performed under the same conditions. After transfection, cell culture medium was changed, and glioblastoma cells were cultured for another 24 h in DMEM plus 10% FBS. Then, cells were applied for Western blot experiments as described below. For controlling the knockdown efficiency, the RNA of transfected cells were purified in parallel with the PicoPure RNA Isolation Kit (Applied Biosystems, Waltham, MA, USA), and qRT-PCR using CXCL16 TaqMan primer probes (Applied Biosystems) was performed as described above. Additionally, cell lysates were also analyzed for CXCL16 protein expression by Western or dot blotting as described below.

### 4.6. Membrane Isolation

For isolation of cell membranes, stable transfected LOX-CXCR6 and LOX-pCMV clone cells were lysed in 5 mM HEPES buffer, then 200 mM HEPES buffer containing 1.4 mM sodium chloride was added, and the mixture was centrifuged at 800 rpm for 8 min at 4 °C. Supernatants were transferred into a new tube and centrifuged once again at 14,000 rpm for 60 min at 4 °C. The remaining pellets were resuspended with 50 µL of 20 mM HEPES buffer including 0.14 mM sodium chloride, and membranes were kept at 4 °C until usage.

### 4.7. Western Blot

Western blotting was performed as described [38]. Briefly, native, sicontrol and siCXCL16 transfected glioblastoma cells, as well as LOX-CXCL16, LOX-pcDNA and truncated LOX-ΔCXCL16 clones were stimulated either with 25 ng/mL recombinant CXCR6 protein (BIOZOL, Eching, Germany), 25 ng/mL recombinant CX3CR1 protein (BIOZOL, as a control) or with 5 µg/mL of LOX-CXCR6 and LOX-pCMV (control) cell membranes for 5–30 min, respectively, and cell lysates were separated by electrophoresis using 10% acrylamide gels. Then, lysates were transferred to polyvinylidene fluoride (PVDF) membranes by blotting, blocked with 5% bovine serum albumin and incubated with a rabbit anti-phospho-ERK1/2 primary antibody (1:1000, Cell Signaling Technology, Danvers, MA, USA, #9101), a rabbit anti-CXCR6 antibody (1:250, Acris, Herford, Germany, SP1286P) or a rabbit anti-CXCL16 antibody (1:250, PeproTech, Hamburg, Germany, #500-P200), and afterwards, the addition of a horseradish-peroxidase labeled secondary antibody (donkey anti-rabbit, Santa Cruz Biotechnology, Santa Cruz, CA, USA) followed by chemo-luminescence detection (GE Healthcare, Munich, Germany or Millipore, Darmstadt, Germany) was performed. To ensure equal loading amounts, membranes were reactivated with methanol, stripped with ReBlot Plus Strong Antibody Strip Solution (Millipore) and re-probed with an antibody against the non-phosphorylated protein (mouse anti-ERK2, 1:200; Santa Cruz Biotechnology, sc1647) or GAPDH (mouse anti-GAPDH, 1:250, Santa Cruz Biotechnology, sc47724). For CXCL16 dot blotting, cell lysates were directly applied to PVDF membranes, blocked, incubated with anti-CXCL16, and signals were detected as described above.

### 4.8. Proliferation Assay

Proliferation assays were performed as described [21]. Briefly, LOX-CXCL16, LOX-pcDNA (1 × 10^5^) and T98G glioblastoma cells (5 × 10^4^) were grown for one day in 10% FBS-supplemented DMEM and stimulated in DMEM plus 0.5% FBS (LOX clones) or 2% FBS (T98G cells) with 50 ng/mL recombinant CXCR6 protein (BIOZOL) for 24 h up to 48 h. In parallel, control groups without stimulation were used. Then, 250 μL of CyQUANT GR dye/cell-lysis buffer and 2.5 μL RNase (CyQUANT^®^ Cell Proliferation Assay Kit (C-7026); Thermo Fisher Scientific, Waltham, MA, USA) were added to the cells, and lysates were scraped off, briefly centrifuged and added 250 µL 2× CyQUANT GR. Sample fluorescence was measured using a fluorescence microplate reader (CM Genios, Tecan, Männedorf, Switzerland) with filters appropriate for 480-nm excitation and 520-nm emission maxima. Results were calculated in ng DNA as the percentage of unstimulated controls.

### 4.9. Anti-Apoptosis Assay

Apoptosis was induced in LOX-CXCL16, LOX-pcDNA and truncated LOX-ΔCXCL16 clones by the addition of 0.1 µg/mL camptothecin (Sigma-Aldrich, St. Louis, MO, USA) applied in a stock solution in DMSO, in the presence or absence of 50 ng/mL recombinant CXCR6 (BIOZOL, Eching, Germany). The final solvent concentration of 0.1% DMSO in camptothecin-treated cultures was also used in controls. After stimulation, cleavage of poly(ADP Ribose) polymerase (PARP) was measured by Western blot (150,000 cells/25 mm^2^ flask, grown for 30 h and stimulated for 18 h) as described above using an antibody specifically detecting cleaved PARP (Asp124, 1:500, Cell Signaling Technology, Danvers, MA, USA). An antibody against GAPDH (1: 500; Santa Cruz Biotechnology, Santa Cruz, CA, USA) served as the loading control.

In T98G glioblastoma cells (250,000 cells/25 mm^2^ flask, grown for 24 h), apoptosis was induced with 400 µg/mL TMZ (solved in DMSO) for 48 h in the presence or absence of 50 ng/mL recombinant CXCR6 (BIOZOL, Eching, Germany). Cells were lysed, and caspase 3/7 activity was measured as previously described [13] and normalized using a caspase 7 standard (Enzo Life Science, Lörrach, Germany).

### 4.10. Migration Assay

Migration was analyzed in wound healing assays (scratch assay; compare [13]). Briefly, 1.5 × 10^5^–1.8 × 10^5^ LOX-CXCL16, LOX-pcDNA, truncated LOX-ΔCXCL16 clones or T98G glioblastoma cells/well were seeded on 6-well dishes, grown to confluence, scratched with a pipet tip, washed and supplemented (or not for controls) with 50 ng/mL recombinant CXCR6 protein (BIOZOL). In each experiment, three scratch regions were photographed at 0 and 8 h. Scratch areas were measured, and differences between 8 and 0 h were determined (yielding the settled area). Stimuli were normalized to non-stimulated controls.

### 4.11. Statistical Analysis

For statistical analysis, a two-tailed Student’s *t*-test was used. Significance levels were *p* < 0.05 (indicated by *), *p* < 0.01 (indicated by **) and *p* < 0.001 (indicated by ***).

## 5. Conclusions

In this study, we could show that the transmembrane chemokine CXCL16 can mediate intracellular signaling upon stimulation with its receptor CXCR6 in the ligand expressing cell. This signaling mechanism has previously been reported for other transmembrane ligands like ephrins, semaphorins and TNF family members and was termed reverse signaling. We now observed that reverse signaling via the transmembrane chemokine CXCL16 promotes migration in the tumor context, but does not affect proliferation or rescue from apoptosis in melanoma or glioblastoma cells. In this line, we could detect that fast migrating glioblastoma cells show higher CXCL16 expression levels in comparison to slowly-migrating cell fractions of the same tumor.

Taken together, being produced as a transmembrane ligand, CXCL16 harbors a broad range of para- and autocrine communication options that may be regulated via expression levels of ligand and receptor (e.g., in inflammation) and via cleavage and release of the chemokine domain by ADAMs. Apart from the classical forward signaling via CXCR6, the transmembrane CXCL16 form may also mediate signaling on its own, either upon binding its soluble CXCL16 (inverse signaling) or upon binding of its receptor CXCR6 (reverse signaling), inducing proliferation and survival, as well as migration in tumor cells.

## Supplementary Materials

Supplementary materials can be found at www.mdpi.com/1422-0067/18/7/1468/s1.

## Abbreviations

ADAM	A disintegrin and metalloproteinase
DMEM	Dulbecco’s Modified Eagle’s Medium
DMSO	Dimethylsulfoxide
ERK	Extracellular signal-regulated kinase (p42/p44)
FBS	Fetal bovine serum
GAPDH	Glyceraldehyde 3-phosphate dehydrogenase
GBM	Glioblastoma multiforme
ICC	Immunocytochemistry
qRT-PCR	Quantitative reverse transcription polymerase chain reaction
