# Supplementary material for: The Chemokine Receptor CXCR6 Evokes Reverse Signaling via the Transmembrane Chemokine CXCL16
Source: Int J Mol Sci. 2017 Jul 8;18(7):1468. doi: 10.3390/ijms18071468 (PMC5535959; doi:10.3390/ijms18071468)
Supplement: Supplementary file 1 [file ijms-18-01468-s001.zip › ijms-203517-proofreading-supplyfile proof.docx]

**Supplementary Materials:**


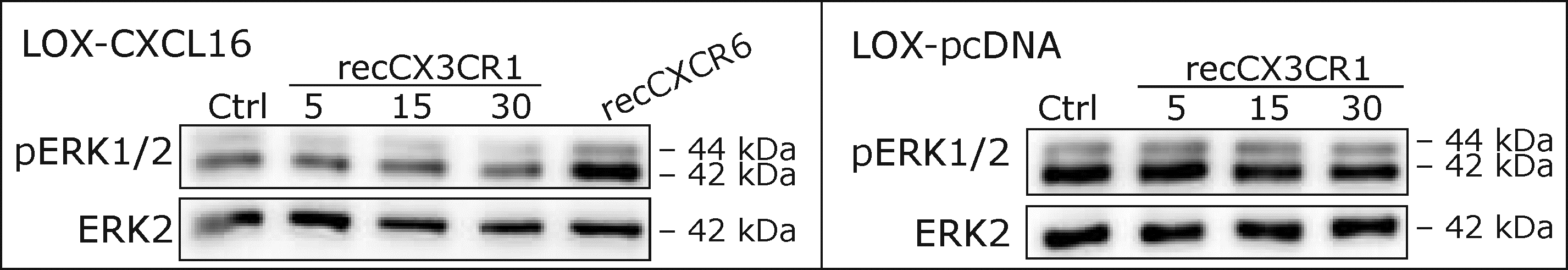


**Figure S1.** To exclude unspecific reactions from recombinant receptors, LOX-CXCL16 and LOX-pcDNA cells were stimulated with recombinant human CX3CR1 (recCX3CR1), an unrelated chemokine receptor, and phosphorylation of ERK1/2 was analyzed by Western blot. RecCX3CR1 stimulation did not activate ERK1/2 signaling in LOX-CXCL16 or LOX-pcDNA cells, while in LOX-CXCL16 a parallel stimulation with recCXCR6 for 15 minutes served as positive control. Examples of n=2 individual experiments are shown.


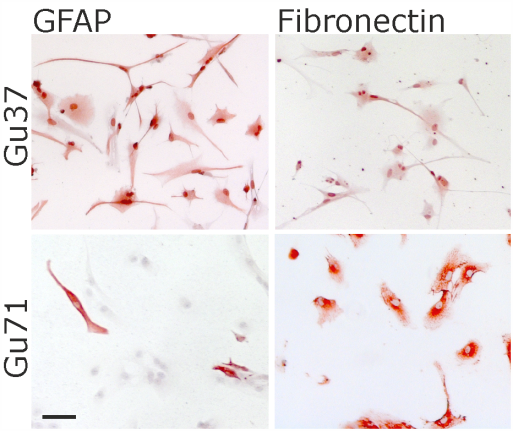


**Figure S2.** Exemplary images of GBM culture routine staining for glial fibrillary acidic protein (GFAP) and fibronectin. Scale bar indicates 50 µm.
