# Supplementary figures and images for: The Chemokine Receptor CXCR6 Evokes Reverse Signaling via the Transmembrane Chemokine CXCL16
Source: Int J Mol Sci. 2017 Jul 8;18(7):1468. doi: 10.3390/ijms18071468 (PMC5535959; doi:10.3390/ijms18071468)

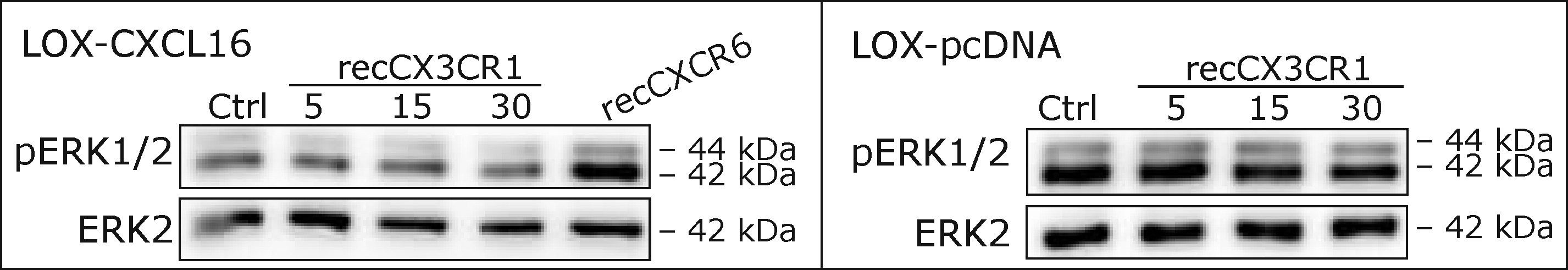

Supplement: Supplementary file 1 [file ijms-18-01468-s001.zip › Proof Supplement figure S1.tif]

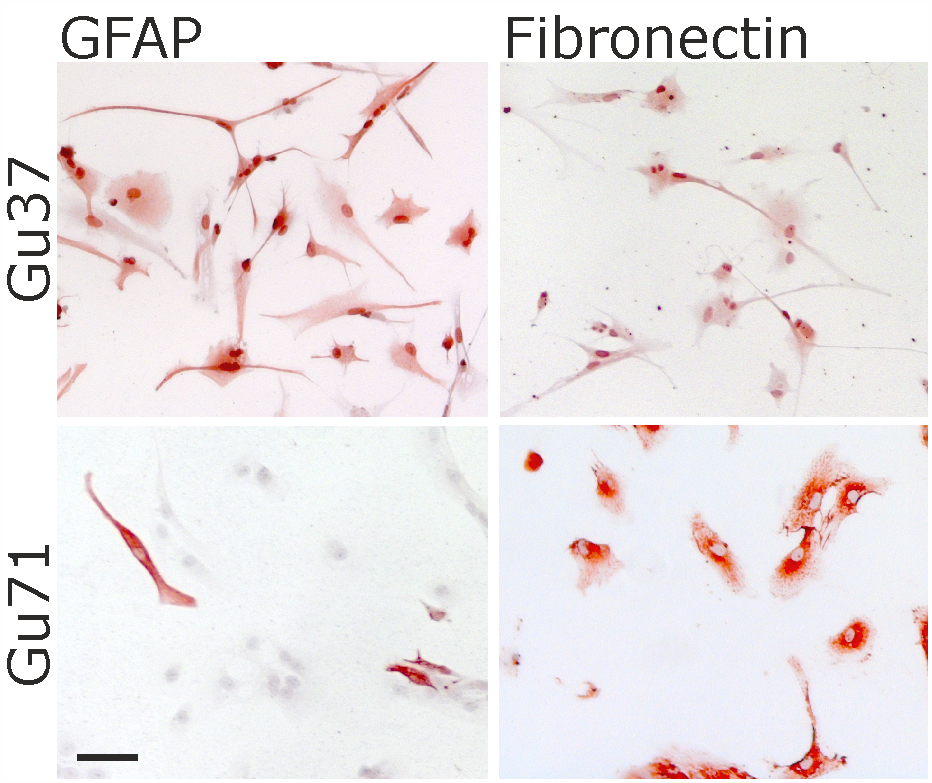

Supplement: Supplementary file 1 [file ijms-18-01468-s001.zip › Proof Supplement figure S2.tif]
